# Development of a secretory expression system with high compatibility between expression elements and an optimized host for endoxylanase production in *Corynebacterium glutamicum*

**DOI:** 10.1186/s12934-019-1116-y

**Published:** 2019-04-17

**Authors:** Wei Zhang, Yankun Yang, Xiuxia Liu, Chunli Liu, Zhonghu Bai

**Affiliations:** 1 National Engineering Laboratory for Cereal Fermentation Technology, Jiangnan University, Wuxi, 214122 China; 2 The Key Laboratory of Industrial Biotechnology, Ministry of Education, School of Biotechnology, Jiangnan University, Wuxi, 214122 China

**Keywords:** *Corynebacterium glutamicum*, Endoxylanase, ClpS, Chromosomal expression, Coexisting plasmids

## Abstract

**Background:**

In terms of protein production, the internal environment of the host influences the activity of expression elements, thus affecting the expression level of the target protein. Native expression elements from a specific strain always function well in the original host. In the present study, to enhance the endoxylanase (XynA) production level in *Corynebacterium glutamicum* CGMCC1.15647 with its native expression elements, approaches to reduce host expression obstacles and to promote expression were evaluated.

**Results:**

We identified the signal peptide of CspB2 in *C. glutamicum* CGMCC1.15647 by MALDI-TOF and applied it along with its promoter for the production of endoxylanase (XynA) in this strain. The native *cspB2* promoter and *cspB2* signal peptide are superior to the well-used *cspB1* promoter and *cspA* signal peptide for XynA expression in *C. glutamicum* CGMCC1.15647, and expression in this strain is superior to the expression in *C. glutamicum* ATCC13032. The highest XynA secretion efficiency level in deep 24-well plates level (2492.88 U/mL) was achieved by disruption of the cell wall protein CspB2 and the protease ClpS, chromosomal integration of *xynA* and coexisting plasmid expression, which increased expression 11.43- and 1.35-fold compared to that of chromosomal expression and pXMJ19-xynA-mediated expression in the original strain, respectively. In fed-batch cultivation, the highest XynA accumulation (1.77 g/L) was achieved in the culture supernatant after 44 h of cultivation.

**Conclusion:**

Adaptation between the expression elements and the host is crucial for XynA production in *C. glutamicum* CGMCC1.15647. Strategies including host optimization, chromosomal integration, and coexistence of plasmids were useful for efficient protein production in *C. glutamicum*.

**Electronic supplementary material:**

The online version of this article (10.1186/s12934-019-1116-y) contains supplementary material, which is available to authorized users.

## Background

In heterologous protein production, the successful production of recombinant proteins is mainly dependent on the interaction between the expression elements and the host. The ideal bacterial host provides a simplified and stable host environment for the function of synthetic biological circuits [1], such as high expression level of target protein, no inclusion body produced, no degradation of the target protein and easy for secretion. Efforts have been made to increase the protein production levels by optimizing the adaptation between the expression elements and the host in *Escherichia coli* [2], *Bacillus subtilis* [3], *Saccharomyces cerevisiae* [4] and *Corynebacterium glutamicum* [5]. Due to the following several characteristics such as (1) no endotoxin production and generally regarded as safe status [6], (2) the ability to secrete properly folded protein into the culture, (3) lack of detectable extracellular protease, *C. glutamicum* is a potential host cell for the secretory production of heterologous proteins, important enzymes and pharmaceutical proteins [7–9]. However, the state-of-the-art of *C. glutamicum* protein production is in its infancy due to its limited genetic tools compared to those for the most-widely used host, *E. coli* [10]. To improve the secretory production level of target proteins in *C. glutamicum*, endogenous expression elements had been explored for protein production in *C. glutamicum* [11, 12]. In addition to the manipulations based on expression elements, the development of a suitable host that is suitable for the expression elements is also important, including decreasing expression barriers and enhancing the factors that facilitate secretion. Deletion of the cell wall component protein CspB and the penicillin-binding protein resulted in high secretion efficiency of an antibody Fab [13]. Protease deletion is one of the way to improve the stability of heterologous protein in host, which can improve the accumulation level of the target protein. For example, disruption of the five protease genes *tppA*, *pepE*, *nptB*, *dpp*IV and *dpp*V showed 34% higher bovine chymosin production than that in double-disruption of *tppA* and *pepE* [14]; the GFP fluorescence intensity was increased by 40.6% in a ClpC disrupted *C. glutamicum* compared to the intensity in wild type [15]. In addition, recombinant protein can also be expressed from a single chromosomal site. Chromosomal expression of GFP and AprE could be 2.9-fold and 1.5-fold-increased compared to that of expression from the common integration site amyE by random knock-in in *B. subtilis* [16]. These cases indicated that host optimization would be a useful method to improve protein production, and we believe these approaches could be attempted to improve recombinant protein expression in *C. glutamicum*.

Endoxylanase (XynA) cleaves the β-1,4-glycosidic bonds present in the main chain of xylan, which is the major constituent of hemicellulose. A better digestibility could be achieved when poultry were fed with the XynA-treaded cereal [17]; addition of XynA could improve the bread volume, reduce stickiness and increase the shelf life [18]; the XynA treated pulp showed superior properties [19, 20]. Reports investigating the secretory production of XynA and its utilization in *S. cerevisiae* and *C. glutamicum* were mainly focused on expression elements [21, 22], and the production level could be increased by improving the complementarity between the host and expression elements.

In the present study, we present an efficient system based on native expression elements for the secretory production of XynA in *C. glutamicum* CGMCCl.15647. In addition to studies on expression elements, the effect of host optimization on the production level of XynA was performed, including cell surface layer protein and protease disruption, integration of the *xynA* gene into the chromosome, and coexisting plasmid expression. Large-scale production of XynA was also performed using the optimized expression system in fed-batch cultivation.

## Results

### Identification of the CspB2 protein of *C. glutamicum* CGMCC1.15647

**Fig. 1:**
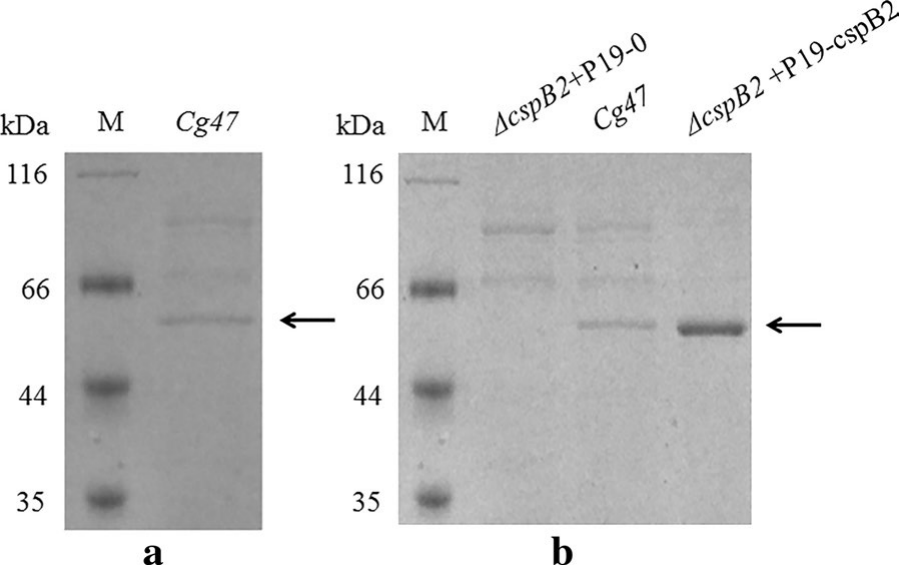
Identification of CspB2 by SDS-PAGE analysis. **a** SDS-PAGE analysis of the proteins in the culture supernatant of *C. glutamicum* CGMCCl.15647. Lane M: protein molecular mass marker; lane *Cg47*: sample from *C. glutamicum* CGMCCl.15647. **b** Identification of CspB2 by SDS-PAGE analysis. Lane M: protein molecular mass marker (*Cg47*: *C. glutamicum* CGMCC1.15647, P19-0: pXMJ19, P19-cspB2, pXMJ19-cspB2). The arrow indicates CspB2

Expression systems based on different backbones, promoters and signal peptides for heterologous protein production in *C. glutamicum* have been summarized [23, 24], and recombination of these expression elements resulted in different expression levels of the same target protein. Considering the heterologous expression elements may not function well in this strain, we wanted to develop an expression system that is suitable for *C. glutamicum* CGMCC1.15647 by using its endogenous expression elements. *C. glutamicum* CGMCC1.15647 was used for EGFP, amylase and ScFv expression in our previous study [25–27]. We believed that the endogenous expression elements from *C. glutamicum* CGMCC1.15647 would be suitable for protein production in that strain. Then, we analyzed the proteins in the culture supernatant of *C. glutamicum* CGMCC1.15647 by SDS-PAGE, and the major protein band at approximately 50 kDa (Fig. 1a) was analyzed by MALDI-TOF. The result showed that this major protein was PS2 (Gi|42560027, PS2, *Corynebacterium glutamicum*) encoded by the *cspB* gene. To distinguish the *cspB* gene from *C. glutamicum* CGMCC1.15647 from other sources, we named it *cspB2*. Primary homology analysis of the region between the *cspB2* promoter, SD sequence and *cspB1* with its SD sequence from *C. glutamicum* ATCC 13869 was performed. The sequences have 91.6% consensus through 501 bp upstream of the start codon, and the − 35 region, − 10 region and SD sequence between the two are the same (Additional file 1: Figure S1A). There were three hydrophobic amino acids that were different between CspB2 and the CspB1 signal peptide: an ‘LVV’ in CspB2 and a ‘VAI’ in CspB1 (Additional file 1: Figure S1B).

To confirm that CspB2 could be expressed and secreted with its promoter and signal peptide at a higher level by plasmid expression than by chromosomal expression, the *cspB2* gene with its signal peptide and promoter was cloned into the pXMJ19 vector and transformed into *ΔcspB2*, which is a CspB2-disrupted strain derived from *C. glutamicum* CGMCC1.15647. The proteins in the culture medium of the transformant were analyzed by SDS-PAGE after cultivation for 48 h. One major protein that matched the molecular weight of CspB2 appeared in *C. glutamicum* CGMCC1.15647 and *ΔcspB2* harboring pXMJ19-cspB2 (Fig. 1b), while the CspB2-disrupted strain *ΔcspB2* lacked this band in the SDS-PAGE gel; we confirmed that this major protein was CspB2 by MALDI-TOF analysis. Figure 1b also shows that the plasmid expression had a higher expression level of CspB2 than did the chromosomal expression in wild-type *C. glutamicum* CGMCC1.15647.

### Secretory production of XynA in *C. glutamicum*

**Fig. 2:**
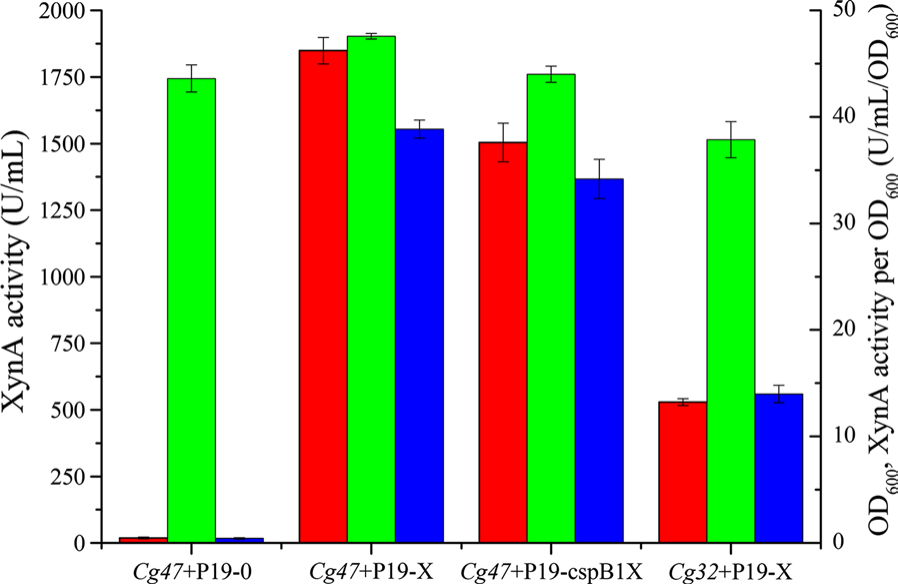
Comparison of the production of XynA using different promoters, signal peptides and hosts. Secretory production level of XynA by *Cg47*+P19-0, *Cg47*+P19-X, *Cg47*+P19-cspB1X and *Cg32*+P19-X. (*Cg32*: *C. glutamicum* ATCC13032, P19-X: pXMJ19-xynA, P19-cspB1X: pXMJ19-cspB1-cspA-xynA). Error bars represent standard deviations. XynA activity (*red*), OD_600_ (*green*), and XynA activity per OD_600_ (*blue*)

XynA plays a key role in the degradation of xylan, one of the most renewable biomass energies in nature. In the present study, we used the endogenous *cspB2* signal peptide with its promoter for XynA production. The wild-type *C. glutamicum* CGMCCl.15647 was transformed with the pXMJ19 and pXMJ19-xynA plasmid to obtain *Cg47*+P19-0 (*C. glutamicum* CGMCCl.15647 carrying pXMJ19) and *Cg47*+P19-X (*C. glutamicum* CGMCCl.15647 carrying pXMJ19-xynA), respectively. The strength of expression from the *cspB2* promoter and *cspB2* signal peptide in *C. glutamicum* CGMCCl.15647 was compared with that from the previously reported *cspB1* promoter and *cspA* signal peptide, which had been used for Fab expression [13], to choose the optimal construct for XynA production. After cultivation for 48 h, the XynA activity in the culture supernatant of *Cg47*+P19-X reached 1849.03 U/mL by the XylX6 kit assay (Fig. 2), which was higher than the production (1504.27 U/mL) of *Cg47*+P19-cspB1X (*C. glutamicum* CGMCCl.15647 carrying pXMJ19-cspB1-cspA-xynA), and there was a higher XynA activity per OD_600_ than that under the *cspB1* promoter and *cspA* signal peptide (Fig. 2). This result indicated that XynA could be successfully expressed and secreted by the endogenous *cspB2* promoter, 5′ UTR and signal peptide of *C. glutamicum* CGMCCl.15647, and the endogenous *cspB2* promoter and *cspB2* signal peptide is superior to the *cspB*1 promoter and *cspA* signal peptide for XynA production in *C. glutamicum* CGMCCl.15647 under the same culture conditions.

To verify the strength of this *cspB2* promoter and signal peptide combination in other *C. glutamicum* subspecies, the pXMJ19-xynA plasmid was transformed into the *C. glutamicum* ATCC 13032 and the resulting strain *Cg32*+P19-X (*C. glutamicum* ATCC 13032 carrying pXMJ19-xynA) was used for XynA production. Under the same culture conditions, the XynA secretion level in strain *Cg32*+P19-X was 528.93 U/mL (Fig. 2), which is only 28.61% of the activity in *Cg47*+P19-X, and the XynA activity per OD_600_ was 35.99% of the activity in *Cg47*+P19-X (Fig. 2).

### Effect of CspB2 and ClpS disruption on XynA secretory expression

**Fig. 3:**
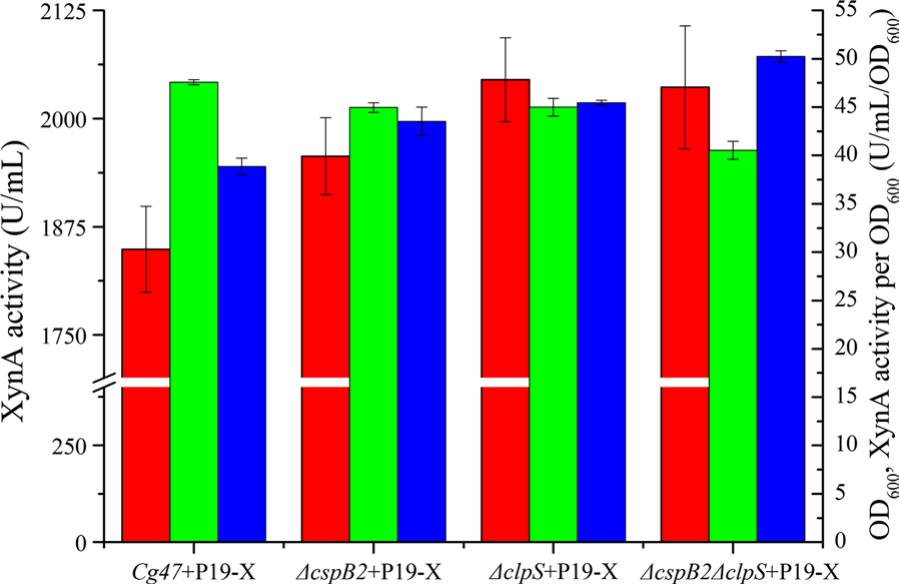
Effect of *ΔcspB2* and *ΔclpS* mutations on XynA production. Secretory production level of XynA by *ΔcspB2*+P19-X, *ΔclpS*+P19-X and *ΔcspB2ΔclpS*+P19-X. Error bars represent standard deviations. XynA activity (*red*), OD_600_ (*green*), and XynA activity per OD_600_ (*blue*)

XynA has been expressed in *C. glutamicum* with different promoters and signal peptides at high levels. Previous authors have mainly focused on plasmid construction, and we believe that the yield of XynA could be further increased by host optimization. The *cspB* gene encodes the S-layer protein CspB, which acts as a cell wall barrier for Fab secretion in ATCC 13869 [13]. The protease ClpS participates in the degradation of unwanted proteins. To improve the productivity of XynA by the *C. glutamicum* CGMCCl.15647 strain, we first investigated the effect of *cspB2* and *clpS* deletion mutations on XynA secretory expression. For this purpose, plasmid pXMJ19-xynA was transformed into *ΔcspB2* and *ΔclpS* to obtain strains *ΔcspB2*+P19-X and *ΔclpS*+P19-X, respectively. In our study, XynA activity reached 1956.59 U/mL in the *cspB2*-disrupted strain *ΔcspB2*+P19-X (Fig. 3), which was 107.56 U/mL greater than the activity in the wild-type strain. It seems that the CspB2 protein forms a physical barrier inhibiting XynA secretion. The *ΔclpS* mutant *ΔclpS*+P19-X increased XynA secreted activity by 195.96 U/mL compared to the activity in wild type (Fig. 3). This is probably due to the ClpS participating in or assisting in the degradation of XynA before secretion.

Both single mutants of *ΔcspB2*+P19-X and *ΔclpS*+P19-X enhanced XynA secretion. To assess the effect of double deletion of *cspB2* and *clpS* on the productivity of XynA, we evaluated the effect of *ΔcspB2* and *ΔclpS* on *C. glutamicum* CGMCCl.15647. However, the secretion level of XynA was 2036.15 U/mL in *ΔcspB2ΔclpS*+P19-X (*ΔcspB2ΔclpS* carrying pXMJ19-xynA) (Fig. 3), indicating that the *cspB2* and *clpS* double mutation had no effect on XynA secretion compared to that of the *clpS* disruption alone (Fig. 3), but the XynA activity per OD_600_ increased 10.5% and 29.3% compared to that of *Cg47*+P19-X and *ΔclpS*+P19-X, respectively (Fig. 3).

### Effect of *xynA* chromosomal integration and coexistence of plasmids on XynA secretion

**Fig. 4:**
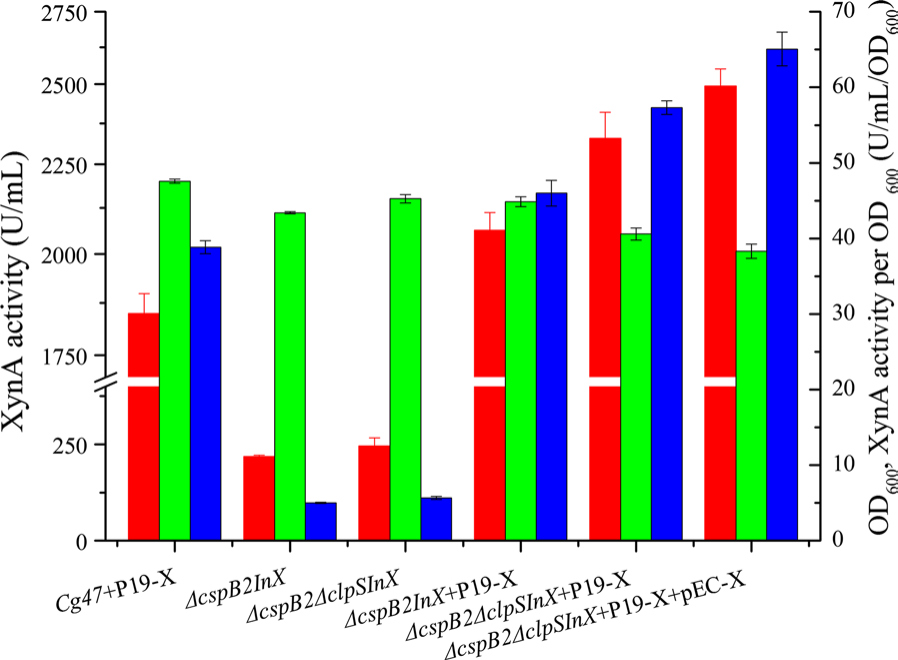
Effect of chromosomal integration and coexistence of plasmids on XynA production. Chromosomal expression of XynA by *ΔcspB2InX* and *ΔcspB2ΔclpSInX*; secretory production of XynA by *ΔcspB2InX*+P19-X, *ΔcspB2ΔclpSInX*+P19-X, and *ΔcspB2ΔclpSInX*+P19-X+pEC-X. (pEC-X: pEC-XK99E-xynA). Error bars represent standard deviations. XynA activity (*red*), OD_600_ (*green*), and XynA activity per OD_600_ (*blue*)

To assess the effect of chromosomal integration of the *xynA* gene on the production of XynA, the *xynA* gene with the *AH6* promoter and *cspB2* signal peptide was integrated into the chromosome of *ΔcspB2* and *ΔcspB2ΔclpS*, yielding *ΔcspB2InX* and *ΔcspB2ΔclpSInX*, respectively. The chromosomal expression of XynA activity in *ΔcspB2InX* and *ΔcspB2ΔclpSInX* was 218.05 and 256.36 U/mL, respectively, which were much lower than that of plasmid expression as expected (Fig. 4). So it is necessary to combine the plasmid expression for further improving the XynA production. When the pXMJ19-xynA plasmid was transformed into *ΔcspB2InX* and *ΔcspB2ΔclpSInX*, the XynA secretion level in the resulting strains *ΔcspB2InX*+P19-X and *ΔcspB2ΔclpSInX*+P19-X achieved 2062.67 and 2327.87 U/mL (Fig. 4), respectively, which were higher than that in the relevant unintegrated mutants *ΔcspB2*+P19-X and *ΔcspB2ΔclpS*+P19-X, and XynA activity per OD_600_ in *ΔcspB2ΔclpSInX*+P19-X was 47.4% higher than that in wild-type *Cg47*+P19-X (Fig. 4). These results indicated that chromosomal integration contributes to XynA expression and further demonstrated that disruption of ClpS had a positive effect on XynA production. Compared to the original *C. glutamicum* CGMCCl.15647 strain, the XynA secretion level in *ΔcspB2ΔclpSInX*+P19-X was increased by 478.84 U/mL.

Generally, multiple plasmids with different replication origins can exist in one host. pXMJ19-xynA and pEC-XK99E-xynA have different antibiotic resistance genes and replicons, so they can coexist in a single *C. glutamicum* cell. To investigate the production of XynA by compatible plasmids in *C. glutamicum*, the plasmids pXMJ19-xynA and pEC-XK99E-xynA were cotransformed into *ΔcspB2ΔclpSInX* to obtain strain *ΔcspB2ΔclpSInX*+P19-X+pEC-X. This coexisting plasmid construct resulted in lower biomass due to the extra burden of the plasmids (Fig. 4); however, highest activity of XynA reached 2492.88 U/mL were present in *ΔcspB2ΔclpSInX*+P19-X+pEC-X (Fig. 4), and the XynA activity per OD_600_ was 67.4% greater than in *Cg47*+P19-X. Finally, the highest yield of XynA was 11.43- and 1.35-fold greater than that for the chromosomal integration expression strain *ΔcspB2InX* and the wild-type *C. glutamicum* CGMCCl.15647 harboring pXMJ19-xynA, respectively, demonstrating that the strategies performed above can be useful for the production of XynA in *C. glutamicum* CGMCCl.15647.

### Enhanced production of XynA by fed-batch cultivation

**Fig. 5:**
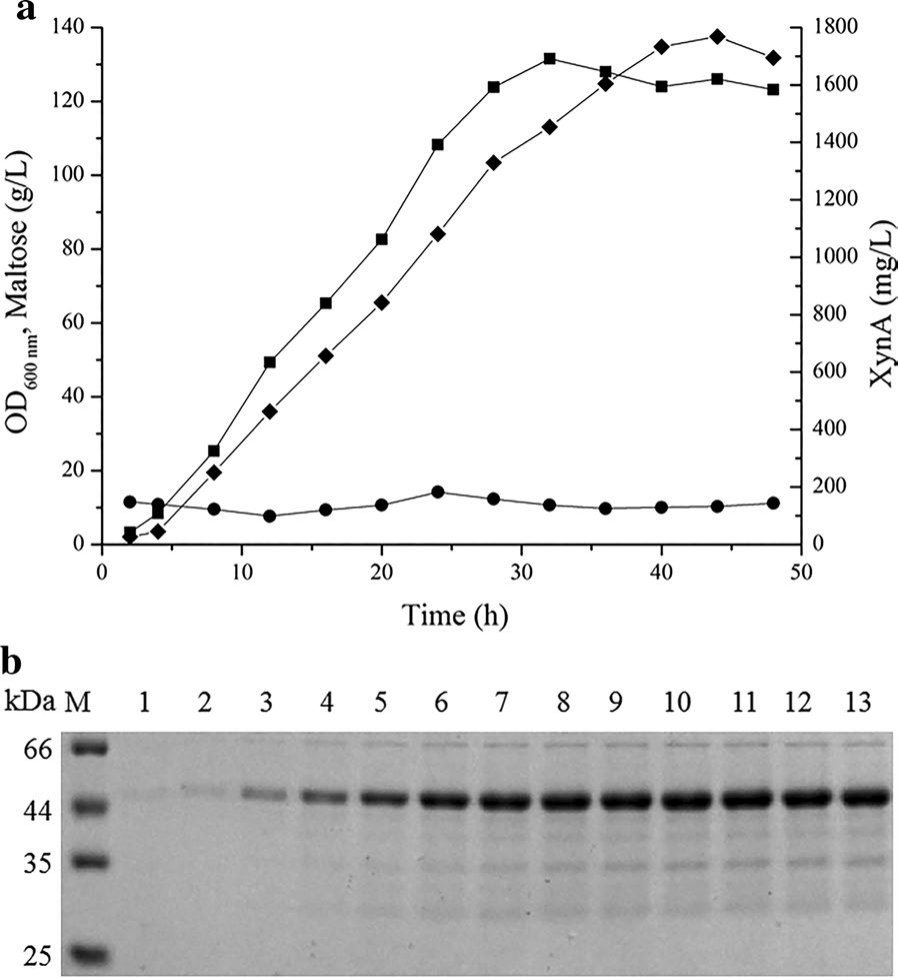
Fed-batch cultivation of *ΔcspB2ΔclpSInX*+P19-X+pEC-X for XynA production. **a** Time profiles of cell growth (square), maltose concentration (circle), and XynA concentration (diamond) in the culture supernatant. **b** SDS-PAGE analysis of the culture supernatant. Lane M: molecular weight markers (kDa); lanes 1–14: samples taken at 2, 4, 8, 12, 16, 20, 24, 28, 32, 36, 40, 44 and 48 h, respectively. Approximately 5 μL culture supernatant was loaded onto each lane

To achieve large-scale production of XynA, fed-batch cultivation with *ΔcspB2ΔclpSInX*+P19-X+pEC-X was carried out in a 5-L bioreactor system. The cells continuously grew to an OD_600_ of 131.52 at 32 h (Fig. 5a) and then decreased gradually. In SDS-PAGE analysis, the XynA band first appeared 8 h after inoculation and increased in density (Fig. 5b). XynA activity could be detected by the XylX6 assay in the culture supernatant at 2 h after inoculation, and it increased quickly in the exponential and stationary phases during cultivation (Fig. 5a). The maximum activity of XynA in the culture supernatant was 3537.24 U/mL at 44 h, which was 12 h after the highest biomass was achieved at 32 h, indicating that XynA accumulated over time. The specific activity of the XynA is 2 U/μg. The highest concentration of XynA that was achieved was 1768.62 mg/L according to the calculation, which was 1.91-fold greater in production yield compared with that of the wild-type *C. glutamicum* CGMCC1.15647 harboring pXMJ19-xynA (924.52 mg/L), and higher than the previously reported 1.54 g/L [28] obtained with a single plasmid in wild-type *C. glutamicum* ATCC 13032. The biomass of our system was not the highest achieved, but we obtained a higher XynA concentration than have previous studies, and the proportion of XynA concentration to OD_600_ was also higher than in previous reports. We believe that the construction of our system for XynA production could be further improved by biomass optimization.

## Discussion

The adaptation between the expression elements and the internal environment of the host is crucial for protein production in synthetic biology. Native expression elements would be preferable for function in the internal environment of a specific strain. Here, we identified the native *cspB2* promoter and *cspB2* signal peptide from *C. glutamicum* CGMCCl.15647 and applied it for XynA expression, and they were superior to the reported *cspB1* promoter and *cspA* signal peptide elements [29]. It seems that the expression level of the target protein is highly related to the suitability of the expression elements for host because there was a much lower XynA expression level in *C. glutamicum* ATCC 13032 than in *C. glutamicum* CGMCC1.15647 with the same expression elements under the same culture conditions.

In addition to plasmid manipulation, another important factor that may affect protein secretion is host optimization, including decreasing barriers that may affect expression and secretion and promoting factors that could facilitate expression and secretion. The XynA expression level could be further increased in *C. glutamicum* CGMCCl.15647 by disrupting the S-layer protein CspB2 and protease ClpS. In *E. coli*, ClpS participates in the recognition of the N-terminus of specific proteins during degradation; ClpS is not essential for degradation, but it enhances degradation [30, 31]. XynA from *Streptomyces coelicolor* A3(2) is a heterologous protein for *C. glutamicum*, and the regulatory system, including the protease, may manipulate this foreign protein.

Moreover, chromosomal integration and coexisting plasmid transformation were performed to facilitate the expression and secretion of XynA. Considering the suitability between expression elements and the host, we chose the native *AH6* promoter and *cspB2* signal peptide of *C. glutamicum* CGMCCl.15647 for *xynA* integration. Coexistence of pXMJ19-xynA and pEC-XK99E-xynA in the optimized *ΔcspB2ΔclpSInX* strain (*ΔcspB2ΔclpSInX*+P19-X+pEC-X) resulted in the highest XynA production, and this coexisting plasmid expression system functioned well because the XynA production level increased with time during Fed-batch cultivation, indicating that coexistence of plasmids would be a useful approach for protein production. Plasmid replication and antibiotic resistance gene expression in coexisting plasmid expression increased the metabolic burden on the host and led to a lower growth rate, and homologous recombination of the *xynA* genes is another increased danger due to the two used plasmids. However, higher expression levels of the target protein could be achieved despite the extra burden or danger. The approaches performed here for XynA production could be useful for improving heterologous protein production in *C. glutamicum*.

## Conclusion

**Fig. 6:**
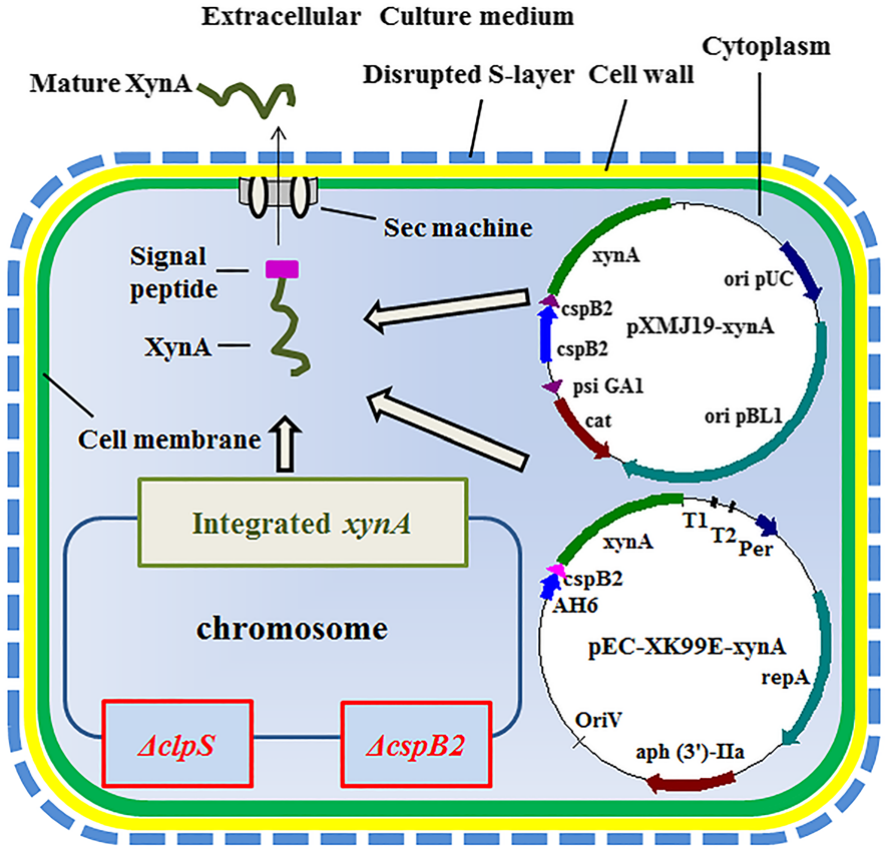
Schematic diagram of optimized *C. glutamicum* CGMCC 1.15647 for the secretory production of XynA. The chromosomal integrated *xynA*, plasmids pXMJ19-xynA and pEC-XK99E-xynA contributed to the XynA production by using the native *AH6* and *cspB2* promoters, and *cspB2* signal peptide. In the ClpS and CspB disrupted mutant, protease could make little influence on the recombinant XynA and XynA secretion could encounter less S-layer barrier after the XynA transporting across the membrane by the Sec machine

In summary, we successfully performed optimization of the secretory production of XynA in *C. glutamicum* by using its native expression elements and further enhanced the production level by chromosomal integration with coexisting plasmids in a cell wall protein (CspB2)- and protease (ClpS)-disrupted mutant (Fig. 6). The highest XynA secretion level reached was 1.77 g/L, which was 14.9% higher than the the recently reported 1.54 g/L [28]. We believe that improvement of the compatibility between the expression elements and the host combined with chromosomal manipulation, not plasmid manipulation alone, will enhance *C. glutamicum* as a potential host for efficient protein production.

## Materials and methods

### Bacterial strains and growth conditions

**Fig. 7:**
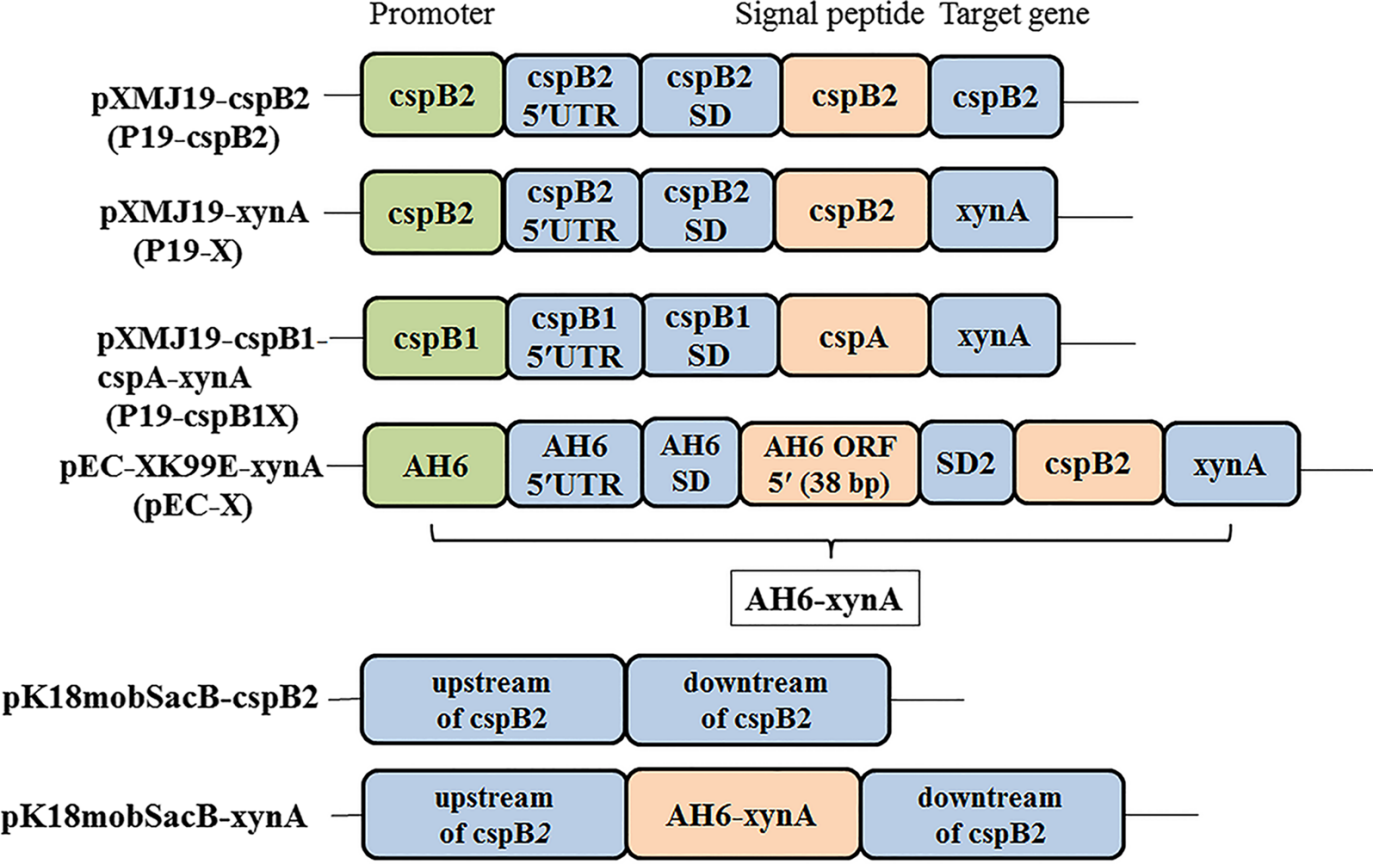
Brief architecture of vectors for protein production and host mutation

The bacterial strains and plasmids used in this study are listed in Table 1 and Fig. 7. *E. coli* was cultivated in LB medium (tryptone, 1 g; yeast extract, 0.5 g; NaCl, 1 g; H_2_O, 100 mL) at 37 °C. *C. glutamicum* ATCC 13032 and *C. glutamicum* CGMCCl.15647 with mutations were cultivated in deep 24-well plates containing 2 mL BHI (brain heart infusion, 37.5 g; H_2_O 1 L) as seed medium. After cultivation at 30 °C for 12 h with shaking at 230 rpm, the seed culture (200 μL) was inoculated into 2 mL MT medium (maltose, 17.61 g; brain heart infusion, 44.87 g; tryptone, 9 g; MgSO_4_, 0.6 g; FeSO_4_·7H_2_O, 1.39 g; biotin, 1 mg; ascorbic acid, 1 mg; riboflavin, 1 mg; ethyl alcohol, 5 mL; H_2_O, 1 L) at 30 °C. The concentrations of chloramphenicol and kanamycin used in *E. coli* and *C. glutamicum* cultures were 30 and 10 μg/mL and 50 and 30 μg/mL, respectively.

**Table 1:** Bacterial strains and plasmids used in this study

Strain or plasmid	Description	Sources
Strains		
*E. coli* DH5α		Lab stock
*C. glutamicum* ATCC13032	Wild type	Lab stock
*C. glutamicum* CGMCCl.15647	Wild type	Lab stock
*ΔcspB2*	*C. glutamicum* CGMCCl.15647, *cspB2* disruption	This work
*ΔclpS*	*C. glutamicum* CGMCCl.15647, *clpS* disruption	Lab stock
*ΔcspB2ΔclpS*	*C. glutamicum* CGMCCl.15647, *cspB2* and *clpS* disruption	This work
*ΔcspB2InX*	*C. glutamicum* CGMCCl.15647, *cspB2* disruption, *xynA* integration	This work
*ΔcspB2ΔclpSInX*	*C. glutamicum* CGMCCl.15647, *cspB2* disruption, *clpS* disruption, *xynA* integration	This work
*Cg47*+P19-0	*C. glutamicum* CGMCCl.15647 harboring pXMJ19	This work
*Cg47*+P19-X	*C. glutamicum* CGMCCl.15647 harboring pXMJ19-xynA	This work
*Cg47*+P19-cspB1X	*C. glutamicum* CGMCCl.15647 harboring pXMJ19-cspB1-cspA-xynA	This work
*Cg32*+P19-X	*C. glutamicum* ATCC13032 harboring pXMJ19-xynA	This work
*ΔcspB2*+P19-X	*ΔcspB2* harboring pXMJ19-xynA	This work
*ΔclpS*+P19-X	*ΔclpS* harboring pXMJ19-xynA	This work
*ΔcspB2ΔclpS*+P19-X	*ΔcspB2ΔclpS* harboring pXMJ19-xynA	This work
*ΔcspB2InX*+P19-X	*ΔcspB2InX* harboring pXMJ19-xynA	This work
*ΔcspB2ΔclpSInX*+P19-X	*ΔcspB2ΔclpSInX* harboring pXMJ19-xynA	This work
*ΔcspB2ΔclpSInX*+P19-X+pEC-X	*ΔcspB2ΔclpSInX* harboring pXMJ19-xynA and pEC-XK99E-xynA	This work
Plasmids		
pXMJ19 (P19-0)	*E. coli*–*C. glutamicum* shuttle vector, Chlr	Lab stock
pXMJ19-cspB2 (P19-cspB2)	pXMJ19 derivative, P_*cspB2*_, *cspB2* signal peptide, *cspB2* gene	
pXMJ19-cspB1-cspA-xynA (P19-cspB1X)	P_*cspB1*_, *cspA* signal peptide, *xynA*	This work
pXMJ19-xynA (P19-X)	pXMJ19 derivative, P_*cspB2*_, *cspB2* signal peptide, *xynA*	This work
pEC-XK99E	*E. coli*–*C. glutamicum* shuttle vector, Kmr	Lab stock
pEC-XK99E-xynA (pEC-X)	pEC-XK99E derivative, P_*AH6*_, *cspB2* signal peptide, *xynA*	This work
pK18mobSacB	*SacB*, suicide vector, Kmr	Lab stock
pK18mobSacB-cspB2	pK18mobSacB derivative, for *cspB2* disruption	This work
pK18mobSacB-xynA	pK18mobSacB derivative, for *xynA* integration	This work

### Plasmid manipulation

The primers used are listed in Table 2. Q5 High-Fidelity DNA Polymerase, Restriction enzymes and T4 DNA ligase were purchased from New England Biolabs (Ipswich, MA, USA). Kits for plasmid and genomic isolation were purchased from Sangon (Shanghai, China). For CspB2 expression, the *cspB2* gene with its promoter was amplified from the *C. glutamicum* CGMCCl.14647 chromosome with the primers cspB2F and cspB2R and ligated to the *Eco*RV and *Eco*RI sites of the pXMJ19 vector by the CloneExpress II One Step Cloning Kit from Vazyme (Nanjing, China), yielding pXMJ19-cspB2. For XynA expression, two plasmids with different backbones, pEC-XK99E-xynA and pXMJ19-xynA, were constructed. The expression vector pEC-XK99E-xynA consists of the *AH6* promoter (Gene ID: NCgl1316), the *cspB2* signal peptide, and the *xynA* gene in pEC-XK99E for XynA expression and was synthesized by Synbio Technologies (Suzhou, China). The pXMJ19-xynA was constructed as follows: the *cspB2* signal peptide with its *cspB2* promoter was PCR-amplified from the genome of *C. glutamicum* CGMCCl.15647 by the primers cspB2F and cspR, the *xynA* gene was PCR-amplified from the pEC-XK99E-xynA clone vector by using the primers xynAF and xynAR, and they were ligated to the *Eco*RV and *Eco*RI sites of the pXMJ19 vector, yielding pXMJ19-xynA. To compare the strength of the *cspB2* promoter and *cspB2* signal peptide of *C. glutamicum* CGMCCl.15647 with that of the *cspB1* promoter and *cspA* signal peptide that had been used for Fab expression [13], the plasmid pXMJ19-*csp*B1-cspA-xynA was synthesized by Synbio Technologies using *xynA* as a reporter gene. pXMJ19-xynA was also transformed into *C. glutamicum* ATCC 13032 to explore the strength of the *cspB2* promoter and the *cspB2* signal peptide in another *C. glutamicum* subspecies. The deletion vector pK18mobSacB-cspB2 for CspB2 knock-out was constructed as follows: the upstream and downstream regions of the *cspB2* gene were PCR-amplified from the *C. glutamicum* CGMCCl.15647 chromosomal DNA template by the primer pairs PS1 + PS2 and PS3 + PS4 and ligated to the *Eco*RI and *Hin*dIII sites of the pK18mobSacB vector, yielding pK18mobSacB-cspB2. The knock-in vector pK18mobSacB-xynA for chromosomal expression of XynA was constructed as follows: the *xynA* gene with the *AH6* promoter and *cspB2* signal peptide was amplified from pEC-XK99E-xynA by using the primers A6XF and A6XR and ligated to the *Xba*I site of pK18mobSacB-cspB2, yielding the *xynA* knock-in vector pK18mobSacB-xynA.

**Table 2:** List of primer oligonucleotide sequences used in this study

Primer	Sequence
cspB2F	cccactaccgagatatccttgaataataattgcaccgcacaggtgatacatg
cspB2R	acagccaagctgaattcttagaacttaacgataccggagaggaatgg
cspR	agtggtttcctgagcgaatgctg
xynAF	tcgctcaggaaaccactgccgagagcacgctcggcgc
xynAR	acagccaagctgaattctcagtggtggtggtggtggtgggtgcgggtccagcgttggttg
PS1	ctatgacatgattacgaattctttatacgtttggttatttgccgactg
PS2	gactctagaggatccccggtggaaccgtcagcgtcgt
PS3	ggggatcctctagagtccgctcagaaggcaatcgctgagg
PS4	acgacggccagtgccaagcttattcggccacgaaggcgccg
A6XF	tccaccggggatcctctagaagatcttcgagctcaagaaggaac
A6XR	gattgccttctgagcggactcagtggtggtggtggtggtgggtgcgggtccagcgttggttg

### Construction of CspB2 and ClpS deletion mutants and XynA integration mutants of *C. glutamicum*

To analyze the effect of host mutation on XynA production, mutations of the cell surface layer protein CspB2 and the protease ClpS were constructed. The protease ClpS (encoded by *clpS*, GenBank: CP025533.1)-disrupted mutant *ΔclpS* was constructed based on homologous recombination in the lab strain. The other mutations were constructed by homologous recombination as described previously [13]. The pK18mobSacB-cspB2 vector was transformed into *C. glutamicum* CGMCCl.15647, the resulting mutant was designated *ΔcspB2* after single-crossover and double-crossover selection. Then, the *cspB2* and *clpS* double mutation was obtained using the same method and designated as *ΔcspB2ΔclpS*. Then, pK18mobSacB-xynA for integration of *xynA* into the chromosome was transformed into *ΔcspB2* and *ΔcspB2ΔclpS*, resulting in the desired *xynA*-integrated mutants *ΔcspB2InX* and *ΔcspB2ΔclpSInX*, respectively. The pXMJ19-xynA and pEC-XK99E-xynA plasmids were then transformed into the *C. glutamicum* mutants alone or together for XynA expression.

### Fed-batch cultivation

*ΔcspB2ΔclpSInX* harboring pXMJ19-xynA and pEC-XK99E-xynA was inoculated into 50 mL BHI as seed medium in a 500-mL flask. After cultivation at 30 °C for 12 h with shaking at 230 rpm, the seed culture (100 mL) was inoculated into 1 L fermentation medium (17.61 g maltose, 44.87 g brain heart infusion, 9 g tryptone, 0.6 g MgSO_4_, 1.39 g FeSO_4_·7H_2_O, 1 mg biotin, 1 mg riboflavin and 1 mg ascorbic acid per liter distilled H_2_O; pH 7.0) in a 5-L jar bioreactor (Applikon EZ-control, Netherlands), and 10 mL ethyl alcohol was added into the jar upon inoculation. The temperature, dissolved oxygen (DO) concentration and the pH were maintained at 30 °C, 35% (v/v) and 7.0, respectively. To prevent glucose starvation, a maltose solution (400 g/L) was added to the culture medium 12 h after inoculation, and 20 mL was added immediately after sampling. Cell growth was monitored by measuring the optical density at 600 nm (OD_600_) with a spectrophotometer.

### Protein preparation and analysis

After cell cultivation in deep plates for 48 h, the culture supernatant was collected by centrifugation at 12,000*g* and 4 °C for 5 min for enzyme activity analysis and SDS-PAGE. To identify the secreted CspB2 protein, the protein band at approximately 50 kDa was excised from the SDS-PAGE gel for the culture supernatant of *C. glutamicum* CGMCCl.15647 and analyzed by MALDI-TOF at Sangon. The activity of XynA was assayed by using the XylX6 kit method (Megazyme, Ireland); the XylX6 assay is highly sensitive and reproducible [32]. One unit of XynA activity is defined as the amount of enzyme required to release 20 nmol of 4-nitrophenol from the XylX6 substrate in 1 min under the defined assay conditions according to the instructions of the XylX6 kit. The protein samples were also analyzed by performing electrophoresis in a 12% (w/v) SDS-PAGE gel. The XynA concentration was measured by using the bicinchoninic acid (BCA) assay.

## Additional file

**Additional file 1: Figure S1.** Comparison of the difference between the *cspB1* and *cspB2* promoters and signal peptides. (A) Nucleic acid base alignment of the *cspB2* and *cspB1* signal peptides using ClustalX software. Identical and similar bases are labeled with asterisks (*). The − 35 region, − 10 region and SD sequence are in the black box. (B) Amino acid sequence alignment of the CspB2 and CspB1 signal peptides using ClustalX software. The different residues are shown in the black box.
