# Supplementary figures and images for: Development of a secretory expression system with high compatibility between expression elements and an optimized host for endoxylanase production in Corynebacterium glutamicum
Source: Microb Cell Fact. 2019 Apr 17;18:72. doi: 10.1186/s12934-019-1116-y (PMC6471998; doi:10.1186/s12934-019-1116-y)

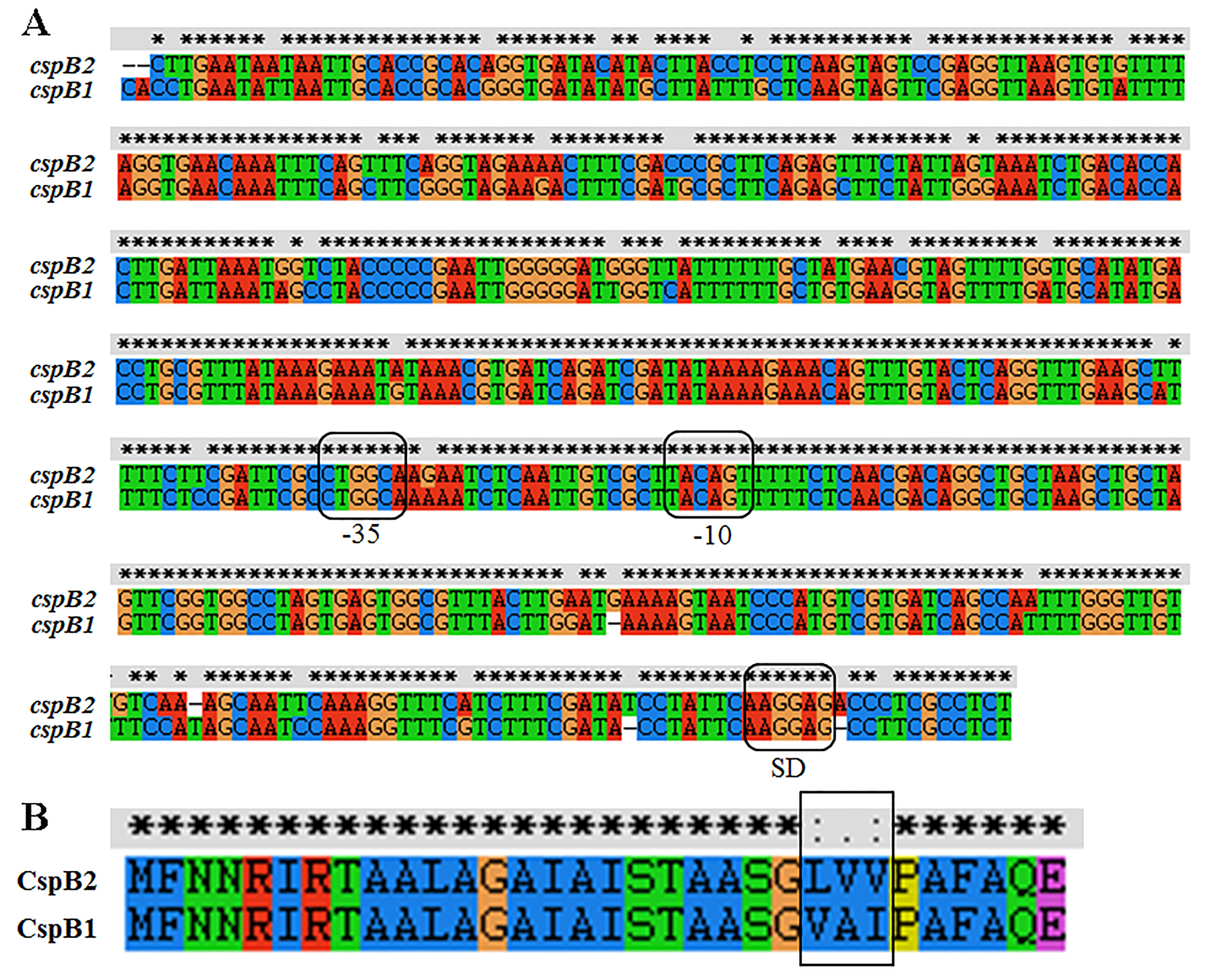

Supplement: Supplementary file 1 — Additional file 1: Figure S1. Comparison of the difference between the cspB1 and cspB2 promoters and signal peptides. (A) Nucleic acid base alignment of the cspB2 and cspB1 signal peptides using ClustalX software. Identical and similar bases are labeled with asterisks (*). The − 35 region, − 10 region and SD sequence are in the black box. (B) Amino acid sequence alignment of the CspB2 and CspB1 signal peptides using ClustalX software. The different residues are shown in the black box. [file 12934_2019_1116_MOESM1_ESM.tif]
